# Systematic review and meta-analysis of the impact of infectious diseases consultation on outcomes of *Staphylococcus aureus* bacteremia in children

**DOI:** 10.1017/ash.2024.450

**Published:** 2024-11-11

**Authors:** Haripriya Santhanam, Nirmal Muthukumarasamy, Mariana Kim Hsieh, Karen Brust, Melanie Wellington, Toshio Naito, Riley J. Samuelson, Alexandre R. Marra, Takaaki Kobayashi

**Affiliations:** 1 Pediatrics, Eastern Iowa Health Center, Cedar Rapids, IA, USA; 2 Department of Internal Medicine, Division of Infectious Diseases, University of Iowa Hospitals and Clinics, Iowa City, IA, USA; 3 The Program of Hospital Epidemiology, University of Iowa Hospitals and Clinics, Iowa City, IA, USA; 4 Department of Pediatrics, Division of Infectious Diseases, University of Iowa Hospitals and Clinics, Iowa City, IA, USA; 5 General Medicine, Juntendo University Hospital, Tokyo, Japan; 6 Hardin Library for the Health Sciences, University of Iowa Libraries, Iowa City, IA, USA; 7 Faculdade Israelita de Ciências da Saúde Albert Einstein, Hospital Israelita Albert Einstein, São Paulo, SP, Brazil; 8 Department of Internal Medicine, Division of Infectious Diseases, University of Kentucky, Lexington, KY, USA

## Abstract

For adult patients with *Staphylococcus aureus* bacteremia (SAB), Infectious Diseases consultation (IDC) significantly lowers mortality and recurrence rate. Our systematic review and meta-analysis demonstrate that IDC is also associated with significantly lower mortality in children with SAB. Analysis of the impact of IDC on pediatric recurrence rates revealed moderate heterogeneity.

## Introduction

*Staphylococcus aureus* is a common cause of both community-acquired and nosocomial bacteremia in children. The incidence of pediatric *S. aureus* bacteremia (SAB) in high-income countries is at 8–26 per 100,000 children per year; 9% of all nosocomial bloodstream infections in children in the USA are due to *S. aureus*.^1^ Mortality in healthy children with SAB ranges from 2%–5% but increases up to 15% in children with pre-existing risk factors such as congenital heart disease.^2^ Several studies evaluating the role of infectious diseases consultation (IDC) in adult patients with SAB have shown a protective effect of IDC on mortality and recurrence rates, as well as improved adherence to quality-of-care metrics. However, there is limited data available regarding the impact of IDC on outcomes of SAB in the pediatric population. The aim of this systematic review and meta-analysis is to assess the impact of IDC on the outcomes of SAB in children.

## Methods

This systematic literature review and meta-analysis was performed in accordance with the Preferred Reporting Items for Systematic Reviews and Meta-Analysis (PRISMA) statement and the Meta-Analysis of Observational Studies in Epidemiology (MOOSE) guidelines. The review protocol was registered on the International Prospective Register for Systematic Reviews (PROSPERO) and is publicly available (CRD 42023473626). Institutional review board approval was not required.

A search strategy to identify publications about SAB and IDC in children was developed in collaboration with a health sciences librarian (RJS) trained in systematic searching (Supplemental Document 1). Systematic strategies were created for PubMed, Embase (Elsevier), Scopus (Elsevier), CINAHL (Ebsco), Cochrane Library (Wiley), and Web of Science (Clarivate). Search terms included controlled vocabulary (when available), synonyms, and related concepts for: *Staphylococcus aureus*, MRSA, bacteremia, septicemia, multi-drug resistant bacteria, pediatrics, children, infectious disease consultation, and antimicrobial stewardship. The searches were conducted in January 2024; no filters were utilized. Additional studies were identified through hand-searching.

Four independent reviewers (H.S., N.M., T.K., and A.R.M.) abstracted data from the studies using a standardized abstraction form. Details of each study were tabulated, including study design, study period, population characteristics, prevalence of methicillin-resistant *Staphylococcus aureus* (MRSA), source of infection, and outcome measures including mortality, SAB recurrence, and fulfillment of certain quality-of-care indicators. H.S. reviewed abstraction forms and served as a tie breaker. One author^3^ was contacted for additional information, which was provided. The Downs and Black scale was used to evaluate the quality of the included studies.

The primary outcomes were all-cause mortality and SAB recurrence rates over any period. We used crude or unadjusted numbers for the pooled odds ratios (ORs) as adjusted ORs were not available in all articles. The pooled OR and 95% confidence interval were calculated using random-effect models with inverse variance weighting. Heterogeneity was assessed with I^2^ estimation. Funnel plots were constructed to assess publication bias. Meta-analyses were conducted using the Cochrane Review Manager (RevMan) version 5.4.

## Results

Among 972 articles screened, a total of 8 studies^2–9^ met the inclusion criteria and were used in the final qualitative systematic literature review (Supplemental Figure 1). All studies evaluated pediatric patients with SAB only. Five^2–6^ of the eight publications directly evaluated the impact of IDC on outcomes in pediatric SAB; results from these studies were pooled for the meta-analysis. Of these 5 studies, 1 was a prospective cross-sectional study^3^, 1 was an observational cohort study^5^, 2 were retrospective cohort studies^2,6^, and 1 was a quasi-experimental study^4^. One study^3^ was a multicenter study across 2 countries, while the other four studies were conducted in a single center (Table 1). The multicenter study^3^ was performed in Australia and New Zealand, two studies^4,6–8^ were performed in the United States, one in Australia^2^, and one in the United Kingdom^5^.

**Figure 1.**
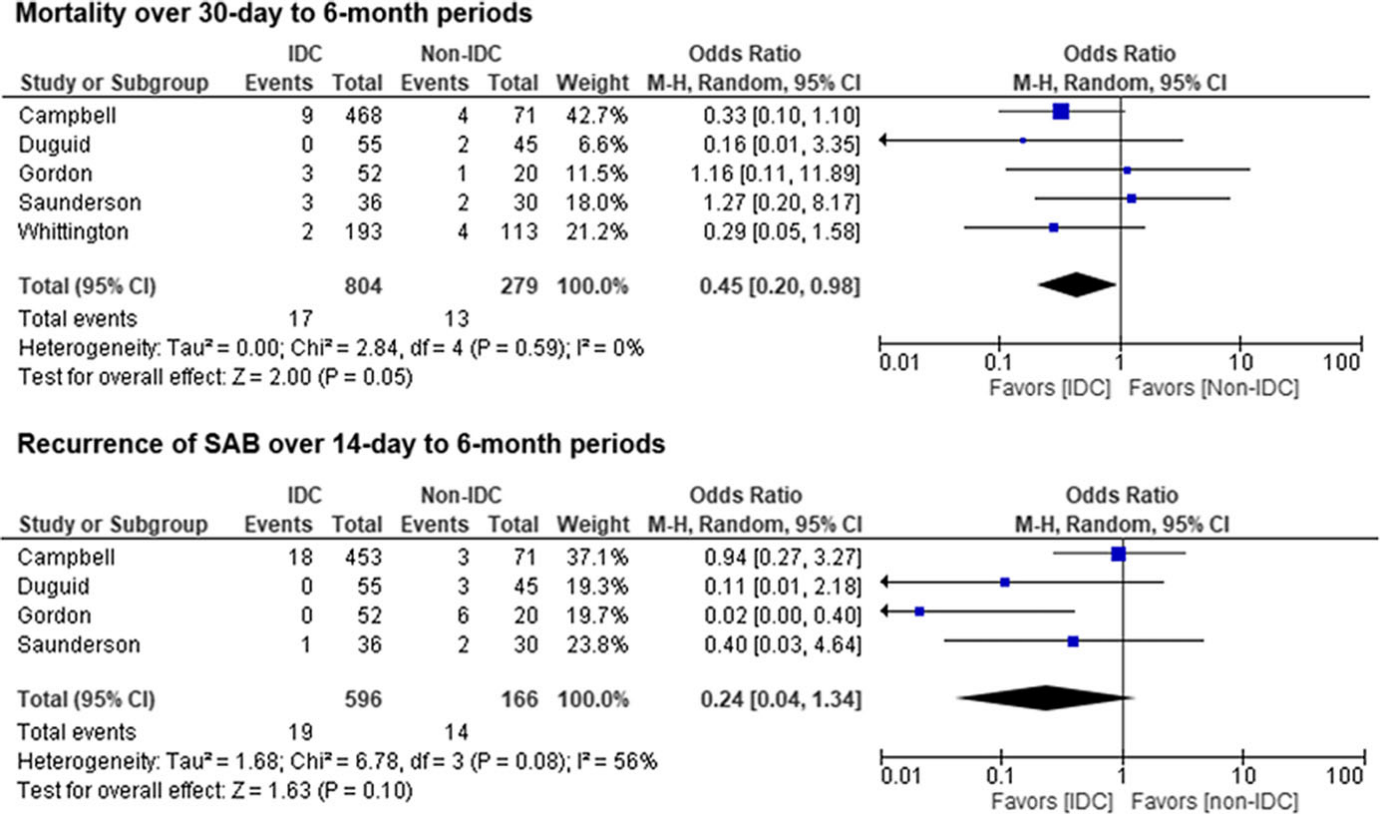
Meta-analysis of outcomes of *Staphylococcus aureus* bacteremia in pediatric patients.

**Table 1.** Summary of the eight studies included in the systematic review

First author/ Publication year/ Location	Setting/ Study period	Study design/ Study objectives/ Adjustment for confounders	Characteristic of included patients/ Prevalence of MRSA/ Source of infection	Outcomes	D&B score
Campbell/2022/ Australia, New Zealand^3^	Multicenter study in 2 countries, including 8 tertiary and 3 secondary pediatric hospitals and 10 NICUs 2 years: 2017–2018	Prospective cross-sectional study Aims were to identify host, pathogen, and treatment factors predictive of the primary outcome, 90-day all-cause mortality, and a composite outcome (defined a priori as 90-day all-cause mortality, 90-day relapse, ICU admission, or length of stay >30 days), in children with SAB Adjustment for confounders: Multivariate regression analysis	Pediatric ≤18 years 24% in-dwelling device 20% ICU admission 9% prematurity 15% immuno-compromised 6% hematologic malignancy 9% congenital heart disease 3% chronic lung disease 17% skin/soft tissue disease 16% MRSA; 79% community-onset bacteremia Source: 49% Osteoarticular 22% Device-related 7% Endovascular focus 15% Skin/soft tissue 14% Pulmonary	90-day all-cause mortality, with IDC as variable, aOR 0.07 (0.004, 0.94), p-value = 0.048 Recurrence of SAB within 90 days was 3.97% in IDC, 4.23% in NIDC, *P* = 0.920	19
Duguid/ 2021/ Sydney, Australia^2^	Single academic center 6 years 5 months: January 2009 to June 2015	Retrospective cohort study Aim to evaluate differences in management and outcomes with and without IDC in children with SAB Outcome measures were rates of cure, relapse, 30-day mortality, median days of fever, bacteremia, hospital stay, median days to source control, and rate of transfer to ICU Adjustment for confounders: Firth multivariate logistic regression analysis	Pediatric ≤18 years 28% malignancy 12% eczema/ dermatitis 0% congenital heart disease 2% prematurity 10% MRSA; 51% community-onset bacteremia Source: 31% Catheter-associated 31% MSK 28% Skin/soft tissue 11% Pulmonary 2% Endocarditis	aOR of cure in those with IDC vs NIDC was 31.5 (1.2, 845.0), P-value 0.04 30-day mortality was 0% in IDC, 4.4% in NIDC, *P* = 0.20 Recurrence of SAB within 14 days was 0% in IDC, 6.7% in NIDC, *P* = 0.09 Differences in management/quality indicators: – Repeat blood cultures 89.1% in IDC vs 75.6% in NIDC, *P* = 0.07 – Echo when indicated 85.7% in IDC vs 66.7% in NIDC, *P* = 0.28 – Source control when indicated 87.5% in IDC vs 19.1% in NIDC, *P* < 0.01 – Switch to directed therapy within 24 hours 98.2% in IDC vs 75.6% in NIDC, *P* < 0.01 – Appropriate choice of directed therapy 98.2% in IDC vs 82.2% in NIDC, *P* < 0.01 – Appropriate dose of directed therapy 98.2% in IDC vs 80.0% in NIDC, *P* < 0.01 – Appropriate duration of antibiotic therapy 94.5% in IDC vs 53.3% in NIDC, *P* < 0.01	22
Gordon/ 2022/ Maryland, USA^4^	Single academic center 3 years: July 2018 through July 2021	Quasi-experimental study QI project aimed at increasing IDC for pediatric patients with SAB through implementation of an electronic advisory comment supporting IDC for SABs Primary outcome measures were to evaluate rates of IDC and SAB recurrence before and after initiating the advisory comment No adjustment for confounders Authors note that ID was typically consulted for the “diagnoses that require treatment beyond 14 days…”	Pediatric 33% ICU admission 15% malignancy 12% cardiac disease 12% pulmonary disease 19% MRSA Source: 51% Catheter-associated 33% Osteoarticular 14% Endovascular 4% Pulmonary	Pre-intervention 61% received IDC Post-intervention 96% received IDC 6-month all-cause mortality 6% in IDC vs 5% in NIDC, *P* = 0.99 SAB recurrence within first 6 months 0% in IDC vs 30% in NIDC, *P* = 0.0002	19
Saunderson/ 2014/ Cambridge, UK^5^	Single academic center 6 years 6 months: Retrospective cohort review from July 2006 to October 2009 (pre-IDC service) Prospective review from November 2009 to December 2012 (with IDC service)	Observational cohort study Aim to evaluate the impact of IDC on management of SAB in children, before and after introduction of an IDC service for SAB in Nov 2009 Outcome measures were duration of hospital admission, death and recurrence of SAB at 30 and 90 days post-bacteremia No adjustment for confounders	Pediatric ≤16 years 54.6% presence of CVC 23.8% neonates 20.6% prematurity 25.4% malignancy 20.6% congenital heart disease 12.7% chronic lung disease 6.4% skin/soft tissue disease 7.6% MRSA; 31.8% community-onset bacteremia Source: 45.5% Catheter-associated 24.2% Skin/soft tissue 24.2% Osteoarticular 3% Thrombophlebitis 3% Pulmonary	30-day mortality 8% in IDC vs 0% in NIDC, *P* = NS 30–90 day mortality 0% in IDC and 6.7% in NIDC, *P* = NS Recurrence of SAB in 30 days 0% in both groups, *P* = NS Recurrence of SAB in 30–90 days 2.8% in IDC vs 6.7% in NIDC, *P* = NS Echo done when indicated 80.6% in IDC vs 33.3% in NIDC, *P* = 0.0001 Focus of infection identified 43.9% in IDC vs 23.3% in NIDC, *P* = 0.003 Source control 95.5% in IDC vs 85.7% in NIDC, *P* = NS Repeat blood cultures obtained 88.9% in IDC vs 86.7% in NIDC, *P* = NS	18
Whittington/ 2022/ Missouri, US^6^	Single academic center 7 years and 4 months: January 2011 to May 2018	Retrospective cohort study Aim to evaluate the impact of pediatric ID consultation on management and outcomes in children with SAB Outcome measures included assessment of adherence to six established quality-of-care indicators (QCIs), and the impact of ID consultation on risk of treatment failure, defined as a composite of all-cause mortality or hospital readmission within 90 days. Adjustment for confounders: Propensity score-weighted analysis and multivariate logistic regression	Pediatric <24 years 50% ICU admission 63% underlying condition (severe prematurity, congenital anomalies, malignancy, cystic fibrosis) 59% complicated bacteremia (fever >72 hours, duration of bacteremia =/> 3 days, metastatic disease, endocarditis) 34% MRSA; 67% community-onset bacteremia Source: 32% Musculoskeletal 23% Catheter-associated 15% Pulmonary 12% Skin/soft tissue 11% Endovascular	63% received IDC Composite outcome of 90-day all-cause mortality or hospital readmission, weighted HR of 1.03 (0.69–1.53) in NIDC Fulfillment of quality-of-care indicator (QCI) measures: – Proof-of-cure blood cultures 95% IDC vs 84% NIDC, *P* = 0.002 – All indicated lab studies 46% IDC vs 8% NIDC, *P* <0.001 – Echo when indicated 32% IDC vs 15% NIDC, *P* = 0.03 – Source control when indicated 57% IDC vs 28% NIDC, *P* < 0.001 – Targeted antibiotic therapy 92% IDC vs 71% NIDC, *P* < 0.001 – Appropriate duration of antibiotic therapy, median of 14 days IDC vs 9 days NIDC, *P* = 0.004 Treatment success was associated with proof-of-cure blood cultures and obtaining all indicated lab studies (93% vs 87% in those with treatment success vs failure, *P* = 0.05 and 39% vs 20% in treatment success vs failure, *P* = 0.001 respectively)	22
Cobos-Carrascosa/2015/ Barcelona, Spain^9^	Single academic center 4 study periods from 1995–2012; in 2005 a specific ASP was implemented for pediatric patients Period1: 1995–1999 Period 2: 2000–2002 Period 3: 2006–2008 Period 4: 2010–2012	Prospective cross-sectional study Aims were to evaluate the changes occur- ring in the incidence, antimicrobial resistance patterns and mortality associated with SAB in pediatric patients over a lengthy study period, during which time ASPs were implemented in the hospital to control these infections. Adjustment for confounders: Multivariate regression	Pediatric <16 years 23% prematurity 23% parenteral nutrition 37% dialysis 8% MRSA; 29% community-onset bacteremia Source: 46.4% Catheter-associated 8.9% Osteoarticular 3.3% Pulmonary	In-hospital all-cause mortality aHR (periods 3 and 4 were following introduction of pediatric-specific ASP): Period 2: 1.27 (0.32–5.04) Period 3: 0.52 (0.92–22.36) Period 4: 0.69 (0.01–13.45), p-value 0.05	17
Lloyd/ 2021/ Michigan, USA^7^	Single academic center 3 years 11 months: Jan 2015-July 2017 (pre-BPA implementation) Aug 2017-Dec 2018 (post-BPA implementation)	Quasi-experimental study Aim was to evaluate the impact of an electronic medical record (EMR)-based best practice advisory (BPA) for pediatric SAB, recommending ID consult and optimal antibiotic therapy based on rapid mecA gene detection. Primary outcome was receipt of ID consult. Secondary outcomes included receipt of optimal therapy, time to ID consult and optimal therapy, recurrent SAB, and 30-day all-cause mortality. Adjustment for confounders: Regression models	Pediatric <21 years 43% ICU admission 21% prematurity 12% malignancy 31% heart disease 25% lung disease 4% skin/soft tissue disease 28.6% MRSA pre-BSA period, 20.7% MRSA post-BPA period; 56% community-onset bacteremia Source: 44% Catheter-associated 10% Endovascular focus 4% Pulmonary 4% Skin/soft tissue 1% Osteoarticular	Comparing pre-BPA to post-BPA study periods, ID consult rates increased from 68.6% to 93.1% (*P* = 0.01), optimal antibiotic therapy was administered in 88.6% vs 96.6% of patients (*P* = 0.28) and median time to optimal antibiotic therapy was reduced from 26.1 hours to 5.5 hours (*P* = 0.03) 1-month all-cause mortality 10.3% in post-BPA period vs 7.1% in pre-BPA period, *P* = 0.43 Recurrent SAB within 1 month 11.5% in post-BPA period vs 3.1% in pre-BPA period, *P* = 0.14; of the 3 patients who had a recurrence in the post-BPA period, two of them were the only two patients in that period who did not receive an ID consult	19
Welch/ 2020/ North Carolina, USA^8^	Single academic medical system 3 years: 2015–2016 (pre-ASP/ RDT) 2017–2018 (post-ASP/ RDT)	Quasi-experimental study The primary objective of this study was to assess the clinical impact of concomitant implementation of an antimicrobial stewardship program (ASP) and rapid diagnostic testing (RDT) in children with SAB. Primary outcome measure was time to optimal antibiotic, secondary outcomes included time to effective antibiotic, antibiotic exposure, duration of bacteremia, length of hospital stay, transfer to the intensive care unit, SAB recurrence, and inpatient mortality. No adjustment for confounders	Pediatric ≤18 years 9% prematurity 1.5% malignancy 12% chronic heart disease 30% MRSA Source: 16% Catheter-associated 1.5% Endovascular focus 10% Skin/soft tissue 50% Osteoarticular 4% Pulmonary	In-hospital all-cause mortality 12% pre-ASP vs 0% post-ASP, *P* = 0.04 Time to optimal therapy 44.3 hours pre-ASP vs 21.3 hours post-ASP, *P* = 0.008 Duration of bacteremia 65.0 hours pre-ASP vs 40.9 hours post-ASP, *P* = 0.03	19

aHR, adjusted hazard ratio; aOR, adjusted odds ratio; ASP, antimicrobial stewardship program; BPA, best practice advisory; ICU, intensive care unit; ID, infectious diseases; IDC, infectious diseases consultation; MRSA, methicillin-resistant S*taphylococcus aureus*; MSK, musculoskeletal; NIDC, no infectious diseases consultation; QI, quality improvement; QCI, quality-of-care indicator; SAB, *Staphylococcus aureus* bacteremia.

Of the remaining three papers included in the qualitative systematic review (Table 1), one^7^ evaluated the impact of a best practice advisory (BPA) recommending IDC and optimal antibiotic therapy based on rapid mecA gene detection and two^8,9^ evaluated the longitudinal impact of implementation of an antimicrobial stewardship program (ASP) on pediatric SAB outcomes. Although these studies did not directly evaluate the impact of IDC on pediatric SAB outcomes, the findings have been summarized and presented in Table 1 to demonstrate the impacts of BPAs and ASPs, which incorporate interdisciplinary infectious diseases expertise, on all-cause mortality, recurrence of SAB and quality-of-care metrics such as choice of and time to optimal antibiotic therapy. Six studies^2–4,6–8^ were considered good (>18 of 28 possible points) per the Downs and Black quality tool while two studies^5,9^ were considered fair (15 – 18 points).

When the results of the five studies^2–6^ were pooled, IDC was associated with significantly lower mortality in pediatric SAB with low heterogeneity (pooled OR = 0.44, 95% confidence interval [CI]: 0.20−0.97, I^2^ = 0%) (Figure 1). When the results of the four studies which reported SAB recurrence rates^2–5^ were pooled, IDC was associated with lower recurrence rates, however this was not statistically significant and there was moderate heterogeneity (pooled OR = 0.24, 95% CI: 0.04−1.34, I^2^ = 56%) (Figure 1). We conducted a publication bias analysis using funnel plots (Supplemental Figure 2). For both analyses, the studies were reasonably balanced around the pooled ORs with little evidence of publication bias.

## Discussion

Our systematic review and meta-analysis revealed a noteworthy association between IDC and lower mortality among children with SAB, showing low heterogeneity. Although patients with IDC tended to have a lower recurrence rate, it did not reach statistical significance and had moderate heterogeneity.

Numerous prior studies have demonstrated the efficacy of IDC among adult patients with bacteremia caused by *Staphylococcus aureus*, Enterococcus species, Candida species, etcetera.^10,11^ Accordingly, for adult patients with SAB, many hospitals in the United States recommend or mandate infectious disease consultations. In contrast, our systematic review identified only five published papers evaluating the utility of IDC in pediatric patients with SAB. The observed benefits in adult studies are likely attributable to appropriateness of antibiotic choice and duration and/or identification of infectious foci^11^. These practices result in optimized antibiotic treatment, definitive source control, and proper follow-up. The studies reviewed demonstrated consistent improvements in quality-of-care metrics for pediatric SAB patients when IDC (Duguid^2^, Saunderson^5^, Whittington^6^) was utilized, as well as with services that incorporate infectious diseases expertise, such as BPAs (Lloyd^7^) and ASPs (Welch^8^), as outlined in Table 1.

This study has limitations. Almost half of the included studies were retrospective and most were not designed to investigate the impact of IDC. The period over which outcomes were evaluated varies substantially across studies, including mortality over 30-day to 6-month periods and recurrence of SAB over 14-day to 6-month periods. Immortal time bias was not specifically evaluated in the included studies. Unmeasured confounders are likely to remain, such as the timing of IDC and/or efficacy of antibiotic selection prior to IDC. Because only 40% of the studies included adjusted odds ratios (OR), we could only use crude numbers to calculate the pooled ORs. Therefore, our calculated ORs do not adjust for confounding factors. Both the incidence and mortality of SAB in pediatric patients was much lower than that for adults. Therefore, a prospective study designed to demonstrate a benefit of IDC in pediatric SAB would necessarily be a very large, multicenter study. Thus, despite the inherent limitations, this meta-analysis of existing data provides an important indicator of the potential value of IDC in children with SAB.

In summary, our study suggests that IDC significantly improves the mortality of pediatric patients with SAB. Prospectively collected clinical information will be needed to help us establish a greater degree of accuracy and to understand the precise mechanisms by which IDC might improve outcomes in children with SAB.

## Supporting information

Santhanam et al. supplementary material Santhanam et al. supplementary material

## Supplementary material

For supplementary material accompanying this paper visit http://doi.org/10.1017/ash.2024.450. click here to view supplementary material
