# Supplementary material for: Systematic review and meta-analysis of the impact of infectious diseases consultation on outcomes of Staphylococcus aureus bacteremia in children
Source: Antimicrob Steward Healthc Epidemiol. 2024 Nov 11;4(1):e199. doi: 10.1017/ash.2024.450 (PMC11574588; doi:10.1017/ash.2024.450)
Supplement: Santhanam et al. supplementary material [file S2732494X24004509sup001.docx]

**Supplemental Figure 1: Literature Search on the Impact of Infectious Disease Consultation in Pediatric Patients with Staphylococcus aureus Bacteremia**


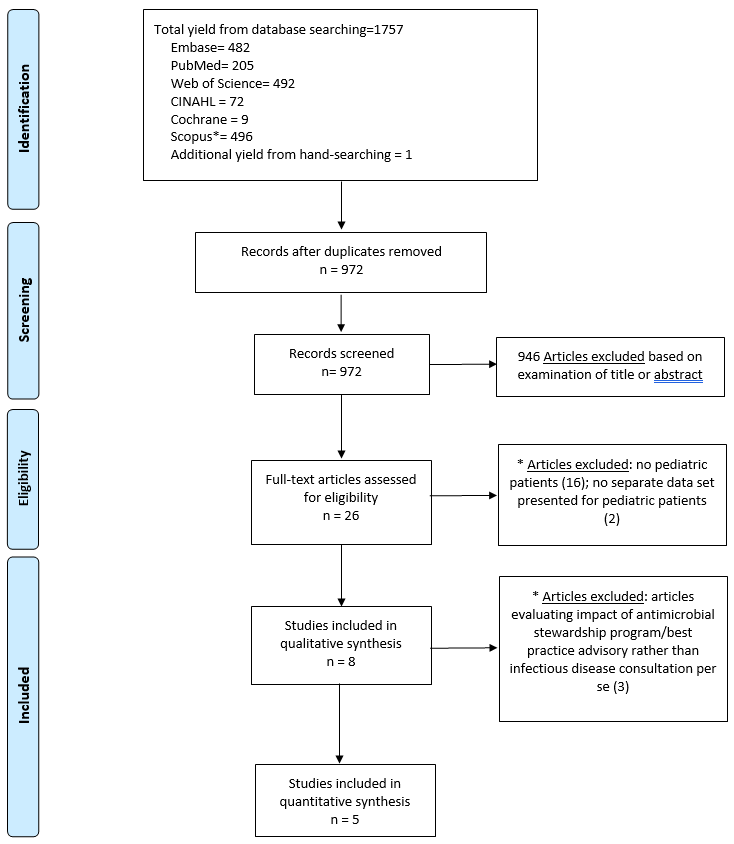


**Supplemental Figure 2: Funnel Plot of Mortality and Recurrence in Pediatric Patients with Staphylococcus aureus Bacteremia**

**
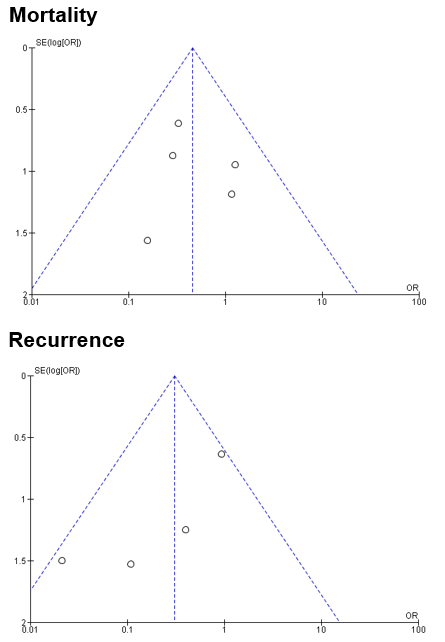
**

**Supplemental Document 1: The search strategies used for our study**

**­PUBMED**

(

**“Staphylococcus aureus”[Mesh] OR**

“aureus”[tiab] OR

“MRSA”[tiab] OR

“VRSA”[tiab]

)

**AND**

(

**"Sepsis”[mesh] OR**

**“**Sepsis”[tiab] OR

“Septicemia*”[tiab] OR

“Septicaemia*”[tiab] OR

“**Bacteremia”[mesh] OR**

**“**Bacteremia*”[tiab] OR

“bacteraemia*”[tiab] OR

“Blood Stream Infection*”[tiab] OR

“bloodstream infection*”[tiab] OR

“positive blood culture*”[tiab] OR

“MDRO*”[tiab] OR

“Multidrug-resistant organism”[tiab] OR

“multidrug resistant bacter*”[tiab] OR

“multiple drug resistant bacter*”[tiab] OR

“multi resistant bacter*”[tiab] OR

“multiresistant bacter*”[tiab]

)

**AND**

(

**"Pediatrics"[Mesh] OR**

“pediat*”[tiab] OR

“paediat*”[tiab] OR

**"Hospitals, Pediatric"[Mesh] OR**

**"Intensive Care Units, Pediatric"[Mesh] OR**

“PICU*”[tiab] OR

“NICU*”[tiab] OR

**"Child"[Mesh] OR**

“child*”[tiab] OR

“Youth*”[tiab] OR

“Juvenile*”[tiab] OR

“toddler*”[tiab] OR

**"Infant"[Mesh] OR**

"Infant*"[tiab] OR

“neonat*”[tiab] OR

“premie*”[tiab] OR

“preemie*”[tiab] OR

“newborn*”[tiab] OR

“baby”[tiab] OR

“babies”[tiab] OR

**"Adolescent"[Mesh] OR**

“Adolescen*”[tiab] OR

“Teen*”[tiab] OR

“youth”[tiab] OR

“youths”[tiab] OR

“kids”[tiab] OR

“kids”[tiab] OR

“boy”[tiab] OR

“boys”[tiab] OR

“girl*”[tiab]

)

**AND**

(

"Referral*”[tiab] OR

“consult*”[tiab] OR

“bundle”[tiab] OR

“Infectious Disease Special*”[tiab] OR

“Infectious Diseases Special*”[tiab] OR

“infection disease special*”[tiab] OR

“infection diseases special*”[tiab] OR

**"Infectious Disease Medicine"[Mesh] OR**

"Infectious Disease Medicine"[tiab] OR

"Infectious Diseases Medicine"[tiab] OR

“infection disease medicine”[tiab] OR

“infection diseases medicine”[tiab] OR

“infectious diseases consult*”[tiab] OR

“infectious disease consult*”[tiab] OR

“infection disease consult*”[tiab] OR

“infection diseases consult*”[tiab] OR

“IDS”[tiab] OR

“IDP”[tiab] OR

“IDC”[tiab] OR

“idcs”[tiab] OR

“idps”[tiab] OR

“idss”[tiab] OR

“ID special*”[tiab] OR

“ID consult*”[tiab] OR

“ID referral*”[tiab] OR

“ID PharmD”[tiab] OR

“Infectious diseases pharmacist*”[tiab] OR

“Infectious disease pharmacist*”[tiab] OR

“best practice advisor*”[tiab] OR

“best practices advisor*”[tiab] OR

“BPA”[tiab] OR

**"Antimicrobial Stewardship"[Mesh] OR**

“antibiotics stewardship”[tiab] OR

“antibiotic stewardship”[tiab] OR

“Antimicrobial Stewardship”[tiab] OR

“Antimicrobials Stewardship”[tiab] OR

“ASP”[tiab] OR

“ASI”[tiab]

)

(**“Staphylococcus aureus”[Mesh] OR** “aureus”[tiab] OR “MRSA”[tiab] OR “VRSA”[tiab])

**AND**

(**"Sepsis”[mesh] OR “**Sepsis”[tiab] OR “Septicemia*”[tiab] OR “Septicaemia*”[tiab] OR “**Bacteremia”[mesh] OR “**Bacteremia*”[tiab] OR “bacteraemia*”[tiab] OR “Blood Stream Infection*”[tiab] OR “bloodstream infection*”[tiab] OR “positive blood culture*”[tiab] OR “MDRO*”[tiab] OR “Multidrug-resistant organism”[tiab] OR “multidrug resistant bacter*”[tiab] OR “multiple drug resistant bacter*”[tiab] OR “multi resistant bacter*”[tiab] OR “multiresistant bacter*”[tiab])

**AND**

(**"Pediatrics"[Mesh] OR** “pediat*”[tiab] OR “paediat*”[tiab] OR **"Hospitals, Pediatric"[Mesh] OR "Intensive Care Units, Pediatric"[Mesh] OR** “PICU*”[tiab] OR “NICU*”[tiab] OR **"Child"[Mesh] OR** “child*”[tiab] OR “Youth*”[tiab] OR “Juvenile*”[tiab] OR “toddler*”[tiab] OR **"Infant"[Mesh] OR** "Infant*"[tiab] OR “neonat*”[tiab] OR “premie*”[tiab] OR “preemie*”[tiab] OR “newborn*”[tiab] OR “baby”[tiab] OR “babies”[tiab] OR **"Adolescent"[Mesh] OR** “Adolescen*”[tiab] OR “Teen*”[tiab] OR “youth”[tiab] OR “youths”[tiab] OR “kids”[tiab] OR “kids”[tiab] OR “boy”[tiab] OR “boys”[tiab] OR “girl*”[tiab])

**AND**

("Referral*”[tiab] OR “consult*”[tiab] OR “bundle”[tiab] OR “Infectious Disease Special*”[tiab] OR “Infectious Diseases Special*”[tiab] OR “infection disease special*”[tiab] OR “infection diseases special*”[tiab] OR **"Infectious Disease Medicine"[Mesh] OR** "Infectious Disease Medicine"[tiab] OR "Infectious Diseases Medicine"[tiab] OR “infection disease medicine”[tiab] OR “infection diseases medicine”[tiab] OR “infectious diseases consult*”[tiab] OR “infectious disease consult*”[tiab] OR “infection disease consult*”[tiab] OR “infection diseases consult*”[tiab] OR “IDS”[tiab] OR “IDP”[tiab] OR “IDC”[tiab] OR “idcs”[tiab] OR “idps”[tiab] OR “idss”[tiab] OR “ID special*”[tiab] OR “ID consult*”[tiab] OR “ID referral*”[tiab] OR “ID PharmD”[tiab] OR “Infectious diseases pharmacist*”[tiab] OR “Infectious disease pharmacist*”[tiab] OR “best practice advisor*”[tiab] OR “best practices advisor*”[tiab] OR “BPA”[tiab] OR **"Antimicrobial Stewardship"[Mesh] OR** “antibiotics stewardship”[tiab] OR “antibiotic stewardship”[tiab] OR “Antimicrobial Stewardship”[tiab] OR “Antimicrobials Stewardship”[tiab] OR “ASP”[tiab] OR “ASI”[tiab])

**2024-01-10 - 205**

**EMBASE**

(

**'Staphylococcus aureus'/exp OR**

**‘**aureus*’:ab,ti,kw OR

‘MRSA’:ab,ti,kw OR

‘VRSA’:ab,ti,kw

)

**AND**

(

**‘Sepsis’/exp OR**

**‘**Sepsis’:ab,ti,kw OR

‘Septicemia*’:ab,ti,kw OR

‘Septicaemia*’:ab,ti,kw OR

‘Severe Sepsis’:ab,ti,kw OR

‘**Bacteremia’/exp OR**

**‘**Bacteremia*’:ab,ti,kw OR

‘bacteraemia*’:ab,ti,kw OR

‘Blood Stream Infection*’:ab,ti,kw OR

‘bloodstream infection*’:ab,ti,kw OR

‘positive blood culture*’:ab,ti,kw OR

**‘multidrug resistant bacterium’/exp OR**

‘multidrug resistant bacteri*’:ab,ti,kw OR

‘MDRO*’:ab,ti,kw OR

‘Multidrug-resistant organism’:ab,ti,kw OR

‘multiple drug resistant bacteri*’:ab,ti,kw OR

‘multi resistant bacter*’:ab,ti,kw OR

‘multiresistant bacter*’:ab,ti,kw

)

**AND**

**(**

**'pediatrics'/exp OR**

‘pediat*’:ab,ti,kw OR

‘paediat*’:ab,ti,kw OR

**'pediatric intensive care unit'/exp OR**

‘PICU*’:ab,ti,kw OR

**'neonatal intensive care unit'/exp OR**

‘NICU*’:ab,ti,kw OR

**'child'/exp OR**

‘child*’:ab,ti,kw OR

‘Youth*’:ab,ti,kw OR

**'juvenile'/exp OR**

‘Juvenile*’:ab,ti,kw OR

‘toddler*’:ab,ti,kw OR

‘Infant*’:ab,ti,kw OR

‘neonat*’:ab,ti,kw OR

‘premie*’:ab,ti,kw OR

‘preemie*’:ab,ti,kw OR

‘newborn*’:ab,ti,kw OR

‘baby’:ab,ti,kw OR

‘babies’:ab,ti,kw OR

**'adolescent'/exp OR**

‘Adolescen*’:ab,ti,kw OR

‘Teen*’:ab,ti,kw OR

‘youth’:ab,ti,kw OR

‘youths’:ab,ti,kw OR

‘kids’:ab,ti,kw OR

‘kids’:ab,ti,kw OR

‘boy’:ab,ti,kw OR

‘boys’:ab,ti,kw OR

‘girl*’:ab,ti,kw

**)**

**AND**

(

‘Referral*’:ab,ti,kw OR

‘consult*’:ab,ti,kw OR

‘bundle’:ab,ti,kw OR

‘Infectious Disease Special*’:ab,ti,kw OR

‘Infectious Diseases Special*’:ab,ti,kw OR

‘infection disease special*’:ab,ti,kw OR

‘infection diseases special*’:ab,ti,kw OR

**‘Infectious Disease Medicine’/exp OR**

‘Infectious Disease Medicine’:ab,ti,kw OR

‘Infectious Diseases Medicine’:ab,ti,kw OR

‘infection disease medicine’:ab,ti,kw OR

‘infection diseases medicine’:ab,ti,kw OR

**'infectious disease specialist'/exp OR**

‘infectious diseases consult*’:ab,ti,kw OR

‘infectious disease consult*’:ab,ti,kw OR

‘infection disease consult*’:ab,ti,kw OR

‘infection diseases consult*’:ab,ti,kw OR

‘IDS’:ab,ti,kw OR

‘IDP’:ab,ti,kw OR

‘IDC’:ab,ti,kw OR

‘idcs’:ab,ti,kw OR

‘idps’:ab,ti,kw OR

‘idss’:ab,ti,kw OR

‘ID special*’:ab,ti,kw OR

‘ID consult*’:ab,ti,kw OR

‘ID referral*’:ab,ti,kw OR

‘ID PharmD’:ab,ti,kw OR

‘Infectious diseases pharmacist*’:ab,ti,kw OR

‘Infectious disease pharmacist*’:ab,ti,kw OR

**'best practice advisory'/exp OR**

‘best practice advisor*’:ab,ti,kw OR

‘best practices advisor*’:ab,ti,kw OR

‘BPA’:ab,ti,kw OR

**'antimicrobial stewardship'/exp OR**

**'antibiotic stewardship program'/exp OR**

‘antibiotics stewardship’:ab,ti,kw OR

‘antibiotic stewardship’:ab,ti,kw OR

‘Antimicrobial Stewardship’:ab,ti,kw OR

‘Antimicrobials Stewardship’:ab,ti,kw OR

‘ASP’:ab,ti,kw OR

‘ASI’:ab,ti,kw

)

(**'Staphylococcus aureus'/exp OR ‘**aureus*’:ab,ti,kw OR ‘MRSA’:ab,ti,kw OR ‘VRSA’:ab,ti,kw)

**AND**

(**‘Sepsis’/exp OR ‘**Sepsis’:ab,ti,kw OR ‘Septicemia*’:ab,ti,kw OR ‘Septicaemia*’:ab,ti,kw OR ‘Severe Sepsis’:ab,ti,kw OR ‘**Bacteremia’/exp OR ‘**Bacteremia*’:ab,ti,kw OR ‘bacteraemia*’:ab,ti,kw OR ‘Blood Stream Infection*’:ab,ti,kw OR ‘bloodstream infection*’:ab,ti,kw OR ‘positive blood culture*’:ab,ti,kw OR **‘multidrug resistant bacterium’/exp OR** ‘multidrug resistant bacteri*’:ab,ti,kw OR ‘MDRO*’:ab,ti,kw OR ‘Multidrug-resistant organism’:ab,ti,kw OR ‘multiple drug resistant bacteri*’:ab,ti,kw OR ‘multi resistant bacter*’:ab,ti,kw OR ‘multiresistant bacter*’:ab,ti,kw)

**AND**

**('pediatrics'/exp OR** ‘pediat*’:ab,ti,kw OR ‘paediat*’:ab,ti,kw OR **'pediatric intensive care unit'/exp OR** ‘PICU*’:ab,ti,kw OR **'neonatal intensive care unit'/exp OR** ‘NICU*’:ab,ti,kw OR **'child'/exp OR** ‘child*’:ab,ti,kw OR ‘Youth*’:ab,ti,kw OR **'juvenile'/exp OR** ‘Juvenile*’:ab,ti,kw OR ‘toddler*’:ab,ti,kw OR ‘Infant*’:ab,ti,kw OR ‘neonat*’:ab,ti,kw OR ‘premie*’:ab,ti,kw OR ‘preemie*’:ab,ti,kw OR ‘newborn*’:ab,ti,kw OR ‘baby’:ab,ti,kw OR ‘babies’:ab,ti,kw OR **'adolescent'/exp OR** ‘Adolescen*’:ab,ti,kw OR ‘Teen*’:ab,ti,kw OR ‘youth’:ab,ti,kw OR ‘youths’:ab,ti,kw OR ‘kids’:ab,ti,kw OR ‘kids’:ab,ti,kw OR ‘boy’:ab,ti,kw OR ‘boys’:ab,ti,kw OR ‘girl*’:ab,ti,kw**)**

**AND**

(‘Referral*’:ab,ti,kw OR ‘consult*’:ab,ti,kw OR ‘bundle’:ab,ti,kw OR ‘Infectious Disease Special*’:ab,ti,kw OR ‘Infectious Diseases Special*’:ab,ti,kw OR ‘infection disease special*’:ab,ti,kw OR ‘infection diseases special*’:ab,ti,kw OR **‘Infectious Disease Medicine’/exp OR** ‘Infectious Disease Medicine’:ab,ti,kw OR ‘Infectious Diseases Medicine’:ab,ti,kw OR ‘infection disease medicine’:ab,ti,kw OR ‘infection diseases medicine’:ab,ti,kw OR **'infectious disease specialist'/exp OR** ‘infectious diseases consult*’:ab,ti,kw OR ‘infectious disease consult*’:ab,ti,kw OR ‘infection disease consult*’:ab,ti,kw OR ‘infection diseases consult*’:ab,ti,kw OR ‘IDS’:ab,ti,kw OR ‘IDP’:ab,ti,kw OR ‘IDC’:ab,ti,kw OR ‘idcs’:ab,ti,kw OR ‘idps’:ab,ti,kw OR ‘idss’:ab,ti,kw OR ‘ID special*’:ab,ti,kw OR ‘ID consult*’:ab,ti,kw OR ‘ID referral*’:ab,ti,kw OR ‘ID PharmD’:ab,ti,kw OR ‘Infectious diseases pharmacist*’:ab,ti,kw OR ‘Infectious disease pharmacist*’:ab,ti,kw OR **'best practice advisory'/exp OR** ‘best practice advisor*’:ab,ti,kw OR ‘best practices advisor*’:ab,ti,kw OR ‘BPA’:ab,ti,kw OR **'antimicrobial stewardship'/exp OR 'antibiotic stewardship program'/exp OR** ‘antibiotics stewardship’:ab,ti,kw OR ‘antibiotic stewardship’:ab,ti,kw OR ‘Antimicrobial Stewardship’:ab,ti,kw OR ‘Antimicrobials Stewardship’:ab,ti,kw OR ‘ASP’:ab,ti,kw OR ‘ASI’:ab,ti,kw)

**2024-01-10 - 482**

**Scopus**

(

**INDEXTERMS(“Staphylococcus aureus”) OR TITLE-ABS-KEY**({aureus} OR {MRSA} OR {VRSA})

)

**AND**

(

**INDEXTERMS(“Sepsis” OR** “**Bacteremia” OR “multidrug resistant bacterium”) OR TITLE-ABS-KEY**(**“**Sepsis” OR “Septicemia*” OR “Septicaemia*” OR “Severe Sepsis” OR **“**Bacteremia*” OR “bacteraemia*” OR “Blood Stream Infection*” OR “bloodstream infection*” OR “positive blood culture*” OR “multidrug resistant bacteri*” OR “MDRO*” OR “Multidrug-resistant organism” OR “multiple drug resistant bacteri*” OR “multi resistant bacter*” OR “multiresistant bacter*”)

)

**AND**

**(**

**INDEXTERMS(“pediatrics” OR “pediatric intensive care unit” OR “neonatal intensive care unit” OR “child” OR “juvenile” OR “adolescent”) OR TITLE-ABS-KEY**( “pediat*” OR “paediat*” OR “PICU*” OR “NICU*” OR “child*” OR “Youth*” OR “Juvenile*” OR “toddler*” OR “Infant*” OR “neonat*” OR “premie*” OR “preemie*” OR “newborn*” OR “baby” OR “babies” OR “Adolescen*” OR “Teen*” OR “youth” OR “youths” OR “kids” OR “kids” OR “boy” OR “boys” OR “girl*”)

**)**

**AND**

(

**INDEXTERMS**(**“Infectious Disease Medicine” OR “infectious disease specialist” OR “best practice advisory” OR “antimicrobial stewardship” OR “antibiotic stewardship program”**) OR TITLE-ABS-KEY(“Referral*” OR “consult*” OR “bundle” OR “Infectious Disease Special*” OR “Infectious Diseases Special*” OR “infection disease special*” OR “infection diseases special*” OR “Infectious Disease Medicine” OR “Infectious Diseases Medicine” OR “infection disease medicine” OR “infection diseases medicine” OR “infectious diseases consult*” OR “infectious disease consult*” OR “infection disease consult*” OR “infection diseases consult*” OR “IDS” OR “IDP” OR “IDC” OR “idcs” OR “idps” OR “idss” OR “ID special*” OR “ID consult*” OR “ID referral*” OR “ID PharmD” OR “Infectious diseases pharmacist*” OR “Infectious disease pharmacist*” OR “best practice advisor*” OR “best practices advisor*” OR “BPA” OR “antibiotics stewardship” OR “antibiotic stewardship” OR “Antimicrobial Stewardship” OR “Antimicrobials Stewardship” OR “ASP” OR “ASI”)

)

**2024-01-10 - 496**

**CINAHL**

(

**(MH "Staphylococcus Aureus+") OR**

(“aureus*”) OR

(“MRSA”) OR

(“VRSA”)

)

**AND**

(

**(MH "Sepsis+") OR**

**(“**Sepsis”) OR

(“Septicemia*”) OR

(“Septicaemia*”) OR

(“Severe Sepsis”) OR

**(MH "Bacteremia") OR**

**(“**Bacteremia*”) OR

(“bacteraemia*”) OR

(“Blood Stream Infection*”) OR

(“bloodstream infection*”) OR

(“positive blood culture*”) OR

(“MDRO*”) OR

(“Multidrug-resistant organism”) OR

(“multidrug resistant bacteri*”) OR

(“multiple drug resistant bacteri*”) OR

(“multi resistant bacter*”) OR

(“multiresistant bacter*”)

)

**AND**

(

**(MH "Pediatrics+") OR**

(“pediat*”) OR

(“paediat*”) OR

**(MH "Hospitals, Pediatric") OR**

**(MH "Intensive Care Units, Pediatric+") OR**

(“PICU*”) OR

(“NICU*”) OR

**(MH "Child+") OR**

(“child*”) OR

(“Youth*”) OR

(“Juvenile*”) OR

(“toddler*”) OR

("Infant*") OR

(“neonat*”) OR

(“premie*”) OR

(“preemie*”) OR

(“newborn*”) OR

(“baby”) OR

(“babies”) OR

**(MH "Adolescence+") OR**

(“Adolescen*”) OR

(“Teen*”) OR

(“youth”) OR

(“youths”) OR

(“kids”) OR

(“kids”) OR

(“boy”) OR

(“boys”) OR

(“girl*”)

)

**AND**

(

**(MH "Referral and Consultation+") OR**

("Referral*”) OR

(“consult*”) OR

(“bundle”) OR

(“Infectious Disease Special*”) OR

(“Infectious Diseases Special*”) OR

(“infection disease special*”) OR

(“infection diseases special*”) OR

(**“**Infectious Disease Medicine”) OR

(“Infectious Diseases Medicine”) OR

(“infection disease medicine”) OR

(“infection diseases medicine”) OR

(“infectious diseases consult*”) OR

(“infectious disease consult*”) OR

(“infection disease consult*”) OR

(“infection diseases consult*”) OR

(“IDS”) OR

(“IDP”) OR

(“IDC”) OR

(“idcs”) OR

(“idps”) OR

(“idss”) OR

(“ID special*”) OR

(“ID consult*”) OR

(“ID referral*”) OR

(“ID PharmD”) OR

(“Infectious diseases pharmacist*”) OR

(“Infectious disease pharmacist*”) OR

(“best practice advisor*”) OR

(“best practices advisor*”) OR

(“BPA”) OR

**(MH "Antimicrobial Stewardship") OR**

(“antibiotics stewardship”) OR

(“antibiotic stewardship”) OR

(“Antimicrobial Stewardship”) OR

(“Antimicrobials Stewardship”) OR

(“ASP”) OR

(“ASI”)

)

(**(MH "Staphylococcus Aureus+") OR** (“aureus*”) OR (“MRSA”) OR (“VRSA”))

**AND**

(**(MH "Sepsis+") OR (“**Sepsis”) OR (“Septicemia*”) OR (“Septicaemia*”) OR (“Severe Sepsis”) OR **(MH "Bacteremia") OR (“**Bacteremia*”) OR (“bacteraemia*”) OR (“Blood Stream Infection*”) OR (“bloodstream infection*”) OR (“positive blood culture*”) OR (“MDRO*”) OR (“Multidrug-resistant organism”) OR (“multidrug resistant bacteri*”) OR (“multiple drug resistant bacteri*”) OR (“multi resistant bacter*”) OR (“multiresistant bacter*”))

**AND**

(**(MH "Pediatrics+") OR** (“pediat*”) OR (“paediat*”) OR **(MH "Hospitals, Pediatric") OR (MH "Intensive Care Units, Pediatric+") OR** (“PICU*”) OR (“NICU*”) OR **(MH "Child+") OR** (“child*”) OR (“Youth*”) OR (“Juvenile*”) OR (“toddler*”) OR ("Infant*") OR (“neonat*”) OR (“premie*”) OR (“preemie*”) OR (“newborn*”) OR (“baby”) OR (“babies”) OR **(MH "Adolescence+") OR** (“Adolescen*”) OR (“Teen*”) OR (“youth”) OR (“youths”) OR (“kids”) OR (“kids”) OR (“boy”) OR (“boys”) OR (“girl*”))

**AND**

(**(MH "Referral and Consultation+") OR** ("Referral*”) OR (“consult*”) OR (“bundle”) OR (“Infectious Disease Special*”) OR (“Infectious Diseases Special*”) OR (“infection disease special*”) OR (“infection diseases special*”) OR (**“**Infectious Disease Medicine”) OR (“Infectious Diseases Medicine”) OR (“infection disease medicine”) OR (“infection diseases medicine”) OR (“infectious diseases consult*”) OR (“infectious disease consult*”) OR (“infection disease consult*”) OR (“infection diseases consult*”) OR (“IDS”) OR (“IDP”) OR (“IDC”) OR (“idcs”) OR (“idps”) OR (“idss”) OR (“ID special*”) OR (“ID consult*”) OR (“ID referral*”) OR (“ID PharmD”) OR (“Infectious diseases pharmacist*”) OR (“Infectious disease pharmacist*”) OR (“best practice advisor*”) OR (“best practices advisor*”) OR (“BPA”) OR **(MH "Antimicrobial Stewardship") OR** (“antibiotics stewardship”) OR (“antibiotic stewardship”) OR (“Antimicrobial Stewardship”) OR (“Antimicrobials Stewardship”) OR (“ASP”) OR (“ASI”))

**2024-01-10 - 72**

**Cochrane**

(

aureus* OR

MRSA OR

VRSA

)

**AND**

(

Sepsis OR

Septicemia* OR

Septicaemia* OR

“Severe Sepsis” OR

Bacteremia* OR

bacteraemia* OR

(“Blood Stream” NEXT Infection*) OR

(bloodstream NEXT infection*) OR

(“positive blood” NEXT culture*) OR

MDRO* OR

“Multidrug-resistant organism” OR

(“multidrug resistant” NEXT bacteri*) OR

(“multiple drug resistant” NEXT bacteri*) OR

(“multi resistant” NEXT bacter*) OR

(multiresistant NEXT bacter*)

)

**AND**

(

pediat* OR

paediat* OR

PICU* OR

NICU* OR

child* OR

Youth* OR

Juvenile* OR

toddler* OR

Infant* OR

neonat* OR

premie* OR

preemie* OR

newborn* OR

baby OR

babies OR

Adolescen* OR

Teen* OR

youth OR

youths OR

kids OR

kids OR

boy OR

boys OR

girl*

)

**AND**

(

Referral* OR

consult* OR

bundle OR

(“Infectious Disease” NEXT Special*) OR

(“Infectious Diseases” NEXT Special*) OR

(“infection disease” NEXT special*) OR

(“infection diseases” NEXT special*) OR

("infection disease” NEXT special*) OR

("infection diseases” NEXT special*) OR

“Infectious Disease Medicine” OR

“Infectious Diseases Medicine” OR

“infection disease medicine” OR

“infection diseases medicine” OR

(“infectious diseases” NEXT consult*) OR

(“infectious disease” NEXT consult*) OR

("infection disease” NEXT consult*) OR

("infection diseases” NEXT consult*) OR

IDS OR

IDP OR

IDC OR

idcs OR

idps OR

idss OR

(ID NEXT special*) OR

(ID NEXT consult*) OR

(ID NEXT referral*) OR

“ID PharmD” OR

(“Infectious diseases” NEXT pharmacist*) OR

(“Infectious disease” NEXT pharmacist*) OR

(“best practice” NEXT advisor*) OR

(“best practices” NEXT advisor*) OR

BPA OR

“antibiotics stewardship” OR

“antibiotic stewardship” OR

“Antimicrobial Stewardship” OR

“Antimicrobials Stewardship” OR

ASP OR

ASI

)

(aureus* OR MRSA OR VRSA)

**AND**

(Sepsis OR Septicemia* OR Septicaemia* OR “Severe Sepsis” OR Bacteremia* OR bacteraemia* OR (“Blood Stream” NEXT Infection*) OR (bloodstream NEXT infection*) OR (“positive blood” NEXT culture*) OR MDRO* OR “Multidrug-resistant organism” OR (“multidrug resistant” NEXT bacteri*) OR (“multiple drug resistant” NEXT bacteri*) OR (“multi resistant” NEXT bacter*) OR (multiresistant NEXT bacter*))

**AND**

(pediat* OR paediat* OR PICU* OR NICU* OR child* OR Youth* OR Juvenile* OR toddler* OR Infant* OR neonat* OR premie* OR preemie* OR newborn* OR baby OR babies OR Adolescen* OR Teen* OR youth OR youths OR kids OR kids OR boy OR boys OR girl*)

**AND**

(Referral* OR consult* OR bundle OR (“Infectious Disease” NEXT Special*) OR (“Infectious Diseases” NEXT Special*) OR (“infection disease” NEXT special*) OR (“infection diseases” NEXT special*) OR ("infection disease” NEXT special*) OR ("infection diseases” NEXT special*) OR “Infectious Disease Medicine” OR “Infectious Diseases Medicine” OR “infection disease medicine” OR “infection diseases medicine” OR (“infectious diseases” NEXT consult*) OR (“infectious disease” NEXT consult*) OR ("infection disease” NEXT consult*) OR ("infection diseases” NEXT consult*) OR IDS OR IDP OR IDC OR idcs OR idps OR idss OR (ID NEXT special*) OR (ID NEXT consult*) OR (ID NEXT referral*) OR “ID PharmD” OR (“Infectious diseases” NEXT pharmacist*) OR (“Infectious disease” NEXT pharmacist*) OR (“best practice” NEXT advisor*) OR (“best practices” NEXT advisor*) OR BPA OR “antibiotics stewardship” OR “antibiotic stewardship” OR “Antimicrobial Stewardship” OR “Antimicrobials Stewardship” OR ASP OR ASI)

**2024-01-10 – 1 CR, 8 Trials**

**Web of Science**

(“aureus*” OR “MRSA” OR “VRSA”)

**AND**

(“Sepsis” OR “Septicemia*” OR “Septicaemia*” OR “Severe Sepsis” OR “Bacteremia*” OR “bacteraemia*” OR “Blood Stream Infection*” OR “bloodstream infection*” OR “positive blood culture*” OR “MDRO*” OR “Multidrug-resistant organism” OR “multidrug resistant bacteri*” OR “multiple drug resistant bacteri*” OR “multi resistant bacter*” OR “multiresistant bacter*”)

**AND**

(“pediat*” OR “paediat*” OR “PICU*” OR “NICU*” OR “child*” OR “Youth*” OR “Juvenile*” OR “toddler*” OR "Infant*" OR “neonat*” OR “premie*” OR “preemie*” OR “newborn*” OR “baby” OR “babies” OR “Adolescen*” OR “Teen*” OR “youth” OR “youths” OR “kids” OR “kids” OR “boy” OR “boys” OR “girl*”)

**AND**

(“Referral*” OR “consult*” OR “bundle” OR “Infectious Disease Special*” OR “Infectious Diseases Special*” OR infection disease special* OR infection diseases special* OR "infection disease special*" OR "infection diseases special*" OR "Infectious Disease Medicine" OR "Infectious Diseases Medicine" OR "infection disease medicine" OR "infection diseases medicine" OR “infectious diseases consult*” OR “infectious disease consult*” OR "infection disease consult*" OR "infection diseases consult*" OR “IDS” OR “IDP” OR “IDC” OR “idcs” OR “idps” OR “idss” OR “ID special*” OR “ID consult*” OR “ID referral*” OR “ID PharmD” OR “Infectious diseases pharmacist*” OR “Infectious disease pharmacist*” OR “best practice advisor*” OR “best practices advisor*” OR “BPA” OR “antibiotics stewardship” OR “antibiotic stewardship” OR “Antimicrobial Stewardship” OR “Antimicrobials Stewardship” OR “ASP” OR “ASI”)

**2024-01-10 – WoS Core Collection, BIOSIS Citation Index, Current Contents Connect, SciELO Citation Index - 492**
